# 2019 Overview

**DOI:** 10.1111/cns.13299

**Published:** 2020-03-03

**Authors:** Cesario Borlongan, Frederic Dorandeu, Giuseppe Di Giovanni, Jian‐Sheng Lin, Gang Hu, Johannes Boltze, Jie Wu, Xiaoming Hu, Yumin Luo

**Affiliations:** ^1^ University of South Florida Tampa FL USA; ^2^ Armed forces biomedical research institute (IRBA) Bretigny‐sur‐Orge France and Ecole du Val de Grace Paris France; ^3^ University of Malta Msida Malta; ^4^ Claude Bernard University Lyon France; ^5^ Nanjing University of Chinese Medicine Nanjing China; ^6^ School of Life Sciences The University of Warwick Coventry UK; ^7^ Barrow Neurological Institute Phoenix AZ USA; ^8^ University of Pittsburgh Pittsburgh PA USA; ^9^ Xuanwu Hospital of Capital Medical University Beijing China

The *CNS Neuroscience & Therapeutics* provides a medium for rapid publication of original clinical, experimental, and translational research papers, timely reviews, and reports of novel findings of therapeutic relevance to the central nervous system. Its focus includes clinical pharmacology, drug development, and novel methodologies for drug evaluation in neurological and psychiatric diseases. We are pleased to announce that *CNS Neuroscience & Therapeutics* has become an Open‐Access Journal as of January 2019. This would allow wider dissemination of scientific knowledge and facilitate collaborative efforts toward advancing novel and solid research on the maintenance of brain homeostasis and repairing the aging and dysfunctional brain.

Over the past year, the Journal covered the latest advancements and future directions in clinical neuroscience with an emphasis on mitochondrial biology, cerebrovascular disease, brain stimulation, and microglia through four special issues. Specifically, the special issue on Mitochondria in Neurodegenerative Diseases highlights how modifiable factors like caloric restriction and exercise can influence mitochondria biogenesis and effective removal following damage, and how disruption of mitochondria quality control mechanisms can lead to neurodegenerative diseases like Parkinson's, Huntington's, and Alzheimer's disease.^1^, ^2^, ^3^ It also updates our current understanding of nonmodifiable factors like genetics and posttranslational modifications on mitochondrial dynamics and their implications in neurological disease. In the special issue on Hemorrhagic Stroke—Pathomechanisms of Injury and Therapeutic Options, we review the molecular changes responsible for direct and secondary brain injury following cerebral hemorrhage and interventions like hyperbaric oxygen preconditioning that may help attenuate these damages.^4^ We also discuss the potential use of magnetic resonance imaging (MRI) as an image‐based biomarker for monitoring treatment and long‐term clinical outcomes of interventions targeting iron‐mediated toxicity. The special issue on Toward Personalized Brain Stimulation: Advances and Challenges highlights the progress of brain stimulation in clinical practice and the mechanisms underlying stimulation‐induced changes in cognitive dysfunction in neurological and psychiatric disorders. Lastly, the special issue on Microglia/Macrophage Diversities in CNS Injuries and Diseases covers various disease‐specific microglia/macrophage profiles. Although their roles in CNS homeostasis and response to neuronal injury have yet to be fully understood, microglia clearly play an integral role in the progression of CNS injuries and disease. Further studies exploring their phenotypic variety and underlying mechanism will hopefully lead to therapeutic strategies that modulate microglia response.

In the coming year, *CNSNT* will have a variety of special issues including Traumatic Brain Injury.

We are delighted to invite proposals for Special Issues on focus topics with high current interest. Recognized experts are encouraged to organize and guest edit special issues with the journal.
